# Poor Readability of Online Patient Resources Regarding Sentinel Lymph Node Biopsy for Melanoma

**DOI:** 10.7759/cureus.3877

**Published:** 2019-01-13

**Authors:** Paul P Yen, Sam M Wiseman

**Affiliations:** 1 Surgery, University of British Columbia, Vancouver, CAN; 2 Surgery, St. Paul’s Hospital & University of British Columbia, Vancouver, CAN

**Keywords:** melanoma, sentinel lymph node biopsy, resources, quality indicators, access to care

## Abstract

Background

Established guidelines recommend that patient educational materials should be set at no higher than a sixth-grade reading level to be considered adequately comprehensible to the general public. Our study objective was to assess the readability of online patient resources related to sentinel lymph node biopsy (SLNB) as part of treatment for melanoma.

Materials and methods

The top 50 results from a Google search (search terms: “sentinel lymph node biopsy melanoma”) were analyzed using seven established readability formulae in order to determine their level of adherence to current guidelines.

Results

We found that the readability of available online patient resources is currently very poor, with only 12% of the websites meeting the sixth-grade reading level criteria according to at least one measure, and 0% meeting the criteria according to all seven assessment tools. Furthermore, half of search results were peer-reviewed academic journal articles not intended for the general public.

Discussion and conclusions

Online patient resources related to SLNB carried out as part of melanoma treatment have poor readability. Several simple measures may be taken in order to make these resources more accessible and comprehensible to a broader audience. These resources should undergo ongoing evaluation, with the ultimate goal being improved readability and patient education.

## Introduction

In recent years, the Internet has become a ubiquitous source of information for patients who are seeking to learn more about their symptoms, conditions, upcoming procedures or any aspect of subjects relevant to their own, or to a friend or family member’s care [1,2]. Powerful internet search engines such as Google are often queried for medical advice well before a patient actually sees a medical professional in consultation [3]. In many ways, an online consultation with “Doctor Google” is understandable due to the appeal of the easily accessible, and seemingly limitless, healthcare-related information the Internet provides. However, while the Internet does have an important role to play in patient education, it is its unfiltered and unvetted nature that makes it imperative that all users carefully scrutinize the accuracy and reliability of the information it provides. Even a resource with a credible author may not be of adequate quality or accessibility for the general public. Readability is a key aspect of accessibility that is often ignored, and guidelines formulated by the American Medical Association (AMA) and the US Department of Health and Human Services (USDHHS), make it clear that patient reading material should be set at no higher than a sixth-grade reading level in order to be considered accessible and comprehensible to the majority of the general public [4]. Readability may be readily evaluated using several validated formulae.

Melanoma, also referred to as malignant melanoma or cutaneous melanoma, is the least common but most lethal form of skin cancer. However, it is amongst the most prevalent cancer types that is diagnosed in young and middle-aged adults, and therefore is responsible for a substantial burden of disease [5]. Early melanoma detection, staging and treatment may also significantly improve patient outcomes. Sentinel lymph node biopsy (SLNB) is a surgical lymph node mapping technique that is commonly utilized in order to identify, for sampling, the first lymph node(s) to which a particular malignancy would metastasize [5]. SLNB also allows for reduction in procedure-related morbidity when compared to the performance of routine lymphadenectomy. It helps the clinician to stage, prognosticate, and select appropriate treatment for melanoma patients [6].

The objective of this study was to assess the readability of online patient resources that are specifically related to SLNB being carried out as part of melanoma treatment, and evaluate these observations within the context of currently established readability guidelines. Furthermore, recommendations that ultimately will make reliable, evidence-based, high-quality and easily understandable health-related information available to a broader audience, will also be reviewed. Several recently published articles have reported that online patient resources for a variety of surgical procedures, and other health conditions including a variety of cancer types, tend to be written at too high of a grade level to allow for adequate comprehensibility [7-14], and we hypothesize that this finding will also hold true for resources related to SLNB that is being performed as part of melanoma treatment.

## Materials and methods

The Google search engine was used to run our search, as it has been reported to be the most prominent and most popular search engine utilized by patients [15]. Prior to conducting the search, the browser data (including cookies and cache) was cleared, and we also made sure it was not logged into any specific user account. The following search terms were entered into a Google Chrome web browser, using the incognito browsing mode: “sentinel lymph node biopsy melanoma”. The search was performed on July 1st, 2017. The top 50 unique web pages yielded from the search that met study inclusion criteria and did not meet study exclusion criteria, were investigated. The study inclusion criteria required the website to be: (1) written completely in English, (2) free-to-access, and (3) specifically contain information regarding SLNB being performed for melanoma. The study exclusion criteria required the website not to: (1) have obvious financial conflicts of interest (i.e., specifically sponsored by an external organization or company, or advertising products for sale), (2) be a news article, or (3) solely be a video.

**Figure 1:**
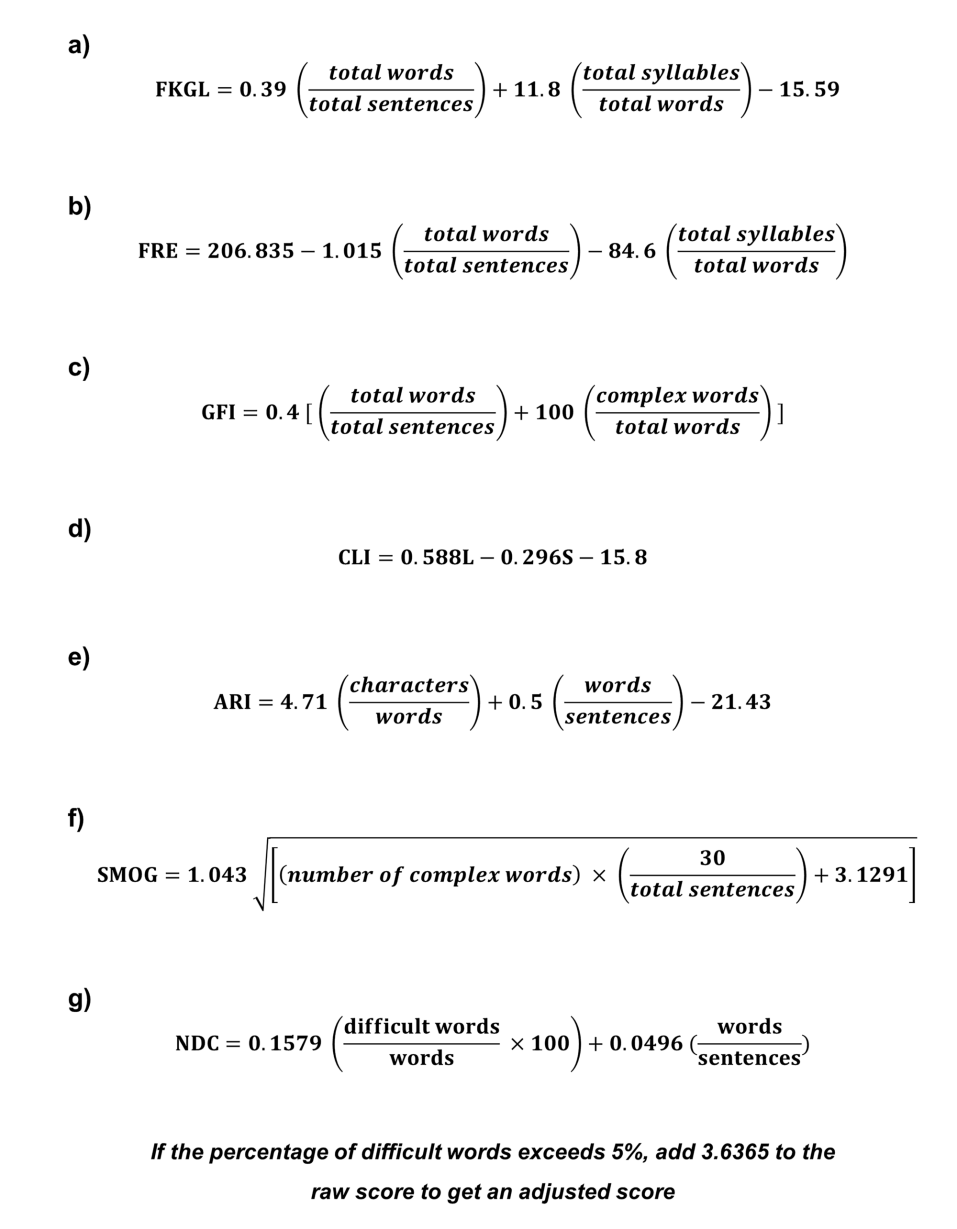
The formulae utilized to calculate the readability measurements in this study. a = Flesch-Kincaid Grade Level; b = Flesch Reading Ease; c = Gunning-Fog Index; d = Coleman-Liau Index; e = Automated Readability Index; f = Simple Measure of Gobbledygook; g = New Dale-Chall Score.

The readability of each website was evaluated using seven different validated readability formulae (Figure 1). The Flesch-Kincaid Grade Level (FKGL) and the Flesch Reading Ease (FRE) both primarily take into account average sentence length, and average syllables per word [16,17]. The Gunning-Fog Score (GFS) is based on average sentence length and the number of polysyllabic words (words containing three or more syllables) [18]. The Coleman-Liau Index (CLI) is based on the variables of average number of letters per 100 words (L), and the average sentence length (S) [19]. The Automated Readability Index (ARI) is similar to the CLI in that it also considers the number of characters per word [20]. The Simple Measure of Gobbledygook (SMOG) index takes three 10-sentence samples near the beginning, middle and end of a piece of text. The number of polysyllabic words are counted and used to calculate a specific grade level. If there are fewer than 30 sentences, the formula includes a correction factor [21]. The New Dale-Chall Score (NDC) includes a list of 3000 words an American fourth-grader can reliably understand, and any word not on this list is considered a “difficult word” [22]. The FRE is a 100-point scale with higher scores indicating more easily understood text. Precise scoring is outlined in Table 1. The FKGL, GFS, CLI, ARI and SMOG indicate the US academic grade level, or number of years of education, that are required in order to comprehend the text. The NDC has a similar but distinct scoring system that is outlined in Table 2.

**Table 1:** Scoring guide for the Flesch Reading Ease. FRE = Flesch Reading Ease; USDHHS = United States Department of Health and Human Services.

FRE Score	Equivalent Education Level	USDHHS Readability
0–29	College Graduate	Difficult
30–49	College	Difficult
50–59	10^th^ to 12^th^ Grade	Difficult
60–69	8^th^ to 9^th ^Grade	Average
70–79	7^th^ Grade	Average
80–89	6^th^ Grade	Easy
90–100	5^th^ Grade	Easy

**Table 2:** Scoring guide for the New Dale-Chall Score. NDC = New Dale-Chall Score.

NDC	Meaning
4.9 or lower	Easily understood by an average 4^th^ grade student or lower
5.0-5.9	Easily understood by an average 5^th ^or 6^th^ grade student
6.0-6.9	Easily understood by an average 7^th ^or 8^th^ grade student
7.0-7.9	Easily understood by an average 9^th ^or 10^th^ grade student
8.0-8.9	Easily understood by an average 11^th ^or 12^th^ grade student
9.0-9.9	Easily understood by an average 11^th ^or 12^th^ grade student

In order to minimize the risk of bias and human error during calculations, and for ease of use, all seven readability tests were administered using a specific online readability calculator recommended by the National Institutes of Health (NIH) (https://readability-score.com/). Prior to analyzing the web pages, the “ideal” readability criteria for online resources were established. The USDHHS recommends health-related materials be written at a sixth-grade level. Thus, for this study, the level of acceptable readability was determined to be greater than or equal to 80.0 for the FRE, less than or equal to 5.9 for the NDC, and less than or equal to 6.9 for the FKGL, GFS, CLI, ARI and SMOG.

Data entry and analyses were performed using a Microsoft® Excel spreadsheet (Microsoft®, Redmond, Washington). Standard independent upper-tailed hypothesis tests were conducted for each readability index, comparing the mean score of websites that were identified in our search with the grade level, or other measure of readability, recommended by the AMA and USDHHS. Differences were considered statistically significant when they yielded a p-value equal to or less than 0.05.

## Results

The first 50 websites yielded by the search that met all of the inclusion criteria, and none of the exclusion criteria, were analyzed for their readability (Table 3). A webpage was categorized as “specialty” if the root website focused on any of the following topics: melanoma, lymph node biopsy, skin cancer, dermatology, or general surgery (10 out of 50); otherwise, it was categorized as “general” (40 out of 50). Moreover, half of the 50 websites were themselves, or contained links to peer-reviewed journal articles, or to guidelines that were derived from them.

**Table 3:** The top 50 websites which were investigated in this study, along with their types (general or specialty) and their readability scores according to all seven formulae. FKGL = Flesch-Kincaid Grade Level; FRE = Flesch Reading Ease; GFI = Gunning-Fog Index; CLI = Coleman-Liau Index; ARI = Automated Readability Index; SMOG = Simple Measure of Gobbledygook; NDC = New Dale-Chall Score.

Website Rank	Website URL	Website Type	FKGL	GFS	CLI	ARI	SMOG	Average Grade Level	FRE	NDC
1	https://www.aimatmelanoma.org/diagnosing-melanoma/sentinel-lymph-node-biopsy/	Specialty	9.2	12.5	10	8.4	12.9	10.6	56.7	6.8
2	http://emedicine.medscape.com/article/854424-overview	General	9.2	12.4	11.9	8.7	12.4	10.9	50.6	8.1
3	http://www.cancer.net/research-and-advocacy/asco-care-and-treatment-recommendations-patients/sentinel-lymph-node-biopsy-melanoma	General	8.9	12.3	10.1	8.7	12.6	10.5	59.4	7
4	http://melanoma.surgery.ucsf.edu/conditions--procedures/sentinel-lymph-node-biopsy.aspx	Specialty	10.1	13.8	11.7	10.6	13.6	12	53.3	8.1
5	http://melanomainternational.org/melanoma-facts/sentinel-node-biopsy/#.WVgyOmjyteU	Specialty	9	12.8	9.6	8.1	12.8	10.5	58.1	6.3
6	https://www.melanoma.org.au/understanding-melanoma/support-patient-stories/patient-support/patient-information-brochures/sentinel-node-biopsy/	Specialty	6.9	10.3	8.2	5.8	10.7	8.4	67.8	5.7
7	https://www.ncbi.nlm.nih.gov/pubmed/19556963	General	16.1	20	13.6	18	17.6	17.1	33.6	8.7
8	https://www.ncbi.nlm.nih.gov/pmc/articles/PMC2879706/	General	12.5	17.7	15.5	13.2	15.8	14.9	34.8	9
9	http://www.skincancer.org/publications/the-melanoma-letter/summer-2015-vol-33-no-1/sentinel	Specialty	14.2	18.3	15.8	15.3	16.6	16	29.8	9
10	http://canjsurg.ca/wp-content/uploads/2014/03/44-6-432.pdf	General	12.9	16.9	13.8	13.1	15.5	14.4	36.8	8.5
11	http://www.cancerresearchuk.org/about-cancer/melanoma/getting-diagnosed/tests-stage/sentinel-lymph-node-biopsy	General	11.6	15.1	13	11.5	14.3	13.1	42.2	8.3
12	https://www.melanoma.org/find-support/patient-community/mpip-melanoma-patients-information-page/newly-diagnosed-do-i-need-sentinel-lymph-node	Specialty	10.8	15.2	12.6	10.2	13.9	12.5	43.1	8
13	http://www.albertahealthservices.ca/assets/info/hp/cancer/if-hp-cancer-guide-cu011-regional-node-dissection.pdf	General	11.5	15.3	11.6	11.1	14.9	12.9	45.6	7.6
14	https://medicine.uiowa.edu/iowaprotocols/sentinel-lymph-node-biopsy	General	11.6	15.5	11.3	11	14.3	12.7	45.9	7.5
15	http://www.cancernetwork.com/review-article/sentinel-lymph-node-biopsy-young-child-thick-cutaneous-melanoma-2	General	13.4	17.8	14	14.1	16.1	15.1	36.6	8.9
16	https://www.hey.nhs.uk/patient-leaflet/sentinel-lymph-node-biopsy-malignant-melanoma/	General	6.4	9.8	8.2	5.9	10.3	8.1	71.1	5.1
17	https://www.intechopen.com/books/melanoma-current-clinical-management-and-future-therapeutics/changing-perceptions-of-lymphadenectomy-and-sentinel-lymph-node-biopsy-in-melanoma	General	8.6	13.2	13.1	9.1	12.1	11.2	51.3	8.3
18	https://surgery.virginia.edu/divisions-of-surgery/division-of-surgical-oncology/melanoma/sentinel-lymph-node-biopsy-for-melanoma/	General	9.9	12.9	8.9	9.2	12.4	10.7	58.2	5.5
19	http://www.cochrane.org/CD010307/SKIN_lymph-node-biopsy-followed-lymph-node-dissection-localised-skin-cancer	General	12.6	13.6	15.7	11.5	13.6	13.6	24.9	9.6
20	http://www.guysandstthomas.nhs.uk/resources/patient-information/dermatology/sentinel-lymph-node-biopsy-for-malignant-melanoma.pdf	General	8.4	12.7	12.9	8.5	11.8	10.9	49.4	8.2
21	http://www.jwatch.org/na33212/2014/01/08/sentinel-lymph-node-biopsies-thin-melanomas	General	13.1	18.1	13.7	12.9	16	14.8	35.3	7.7
22	http://www.futuremedicine.com/doi/abs/10.2217/ebo.13.71	General	11	14.6	13	11	14	12.7	44.5	8.4
23	http://www.melanomahopenetwork.org/Sentinel-Lymph-Node-Biopsy/	Specialty	5.7	9.3	7	4	10	7.2	71	4.9
24	http://www.pathologyoutlines.com/topic/skintumormelanocyticsentinelmelanoma.html	General	11.8	15.9	13.4	12	14.9	13.6	41.1	8.5
25	http://www.gotoper.com/publications/ajho/2014/2014aug/sentinel-lymph-node-biopsy-in-melanoma-and-other-cutaneous-malignancies	General	11.7	16.2	14.5	11.7	14.6	13.7	37.7	8.9
26	http://www.mayoclinic.org/tests-procedures/sentinel-node-biopsy/multimedia/sentinel-node-biopsy/vid-20084727	General	8.9	12.4	9.9	8.7	12.3	10.4	60.4	5.3
27	http://jamanetwork.com/journals/jamadermatology/fullarticle/190100	Specialty	10.3	13.4	12.2	9.9	13.5	11.9	47.5	7.9
28	http://www.racgp.org.au/afp/2014/july/sentinel-lymph-node-biopsy/	General	7.7	10.9	11.9	7.7	11.6	10	54.2	9
29	https://www.guideline.gov/summaries/summary/37870	General	6.5	9.5	9.8	7	10.8	8.7	69.3	6.7
30	https://www.cancer.org/cancer/melanoma-skin-cancer/detection-diagnosis-staging/how-diagnosed.html	General	10.9	15.1	11.9	10	14.3	12.4	44.3	7.6
31	http://www.cancercenter.com/melanoma/sentinel-lymph-node-biopsy/	General	12.8	17.6	12	12.7	15.9	14.2	41.7	8.7
32	https://www.cancercare.on.ca/common/pages/UserFile.aspx?fileId=73874	General	12.8	16.8	13.1	13.3	15.7	14.3	40.6	7.9
33	http://www.patientresource.com/melanoma_lymph_node_mapping.aspx	General	7	10.5	9.2	6.9	11	8.9	67.9	5.4
34	http://clincancerres.aacrjournals.org/content/clincanres/12/7/2320s.full.pdf	General	10.4	14.8	14.3	10.5	13.6	12.7	41.8	9.3
35	http://www.scielo.br/scielo.php?script=sci_arttext&pid=S0102-86502007000500002&lng=en&nrm=iso&tlng=en	General	12.3	16.5	14.1	13.5	15.3	14.3	42	8.5
36	https://am.asco.org/daily-news/use-sentinel-lymph-node-biopsy-patients-melanoma	General	10.5	14.4	13.2	11.1	13.8	12.6	48	8.5
37	http://www.mdedge.com/ccjm/article/99096/dermatology/reply-role-sentinel-lymph-node-biopsy-after-excision-melanomas	General	8.8	13.1	12.8	8.6	12	11.1	48.2	9.6
38	http://www.jaad.org/article/S0190-9622(06)00628-1/fulltext	Specialty	10.5	15	13.3	10.3	13.8	12.6	44	8.1
39	http://www.turner-white.com/pdf/hp_jan00_lymph.pdf	General	12.9	17.8	14.7	13.5	16	15	35.2	9.2
40	https://www.hindawi.com/journals/crionm/2013/259326/	General	11.8	17.2	15	11.4	14.9	14.1	33.2	9.6
41	https://www.karger.com/Article/Pdf/381719	General	11	15	13.8	11	14	13	14.5	8.4
42	https://www.dovepress.com/sentinel-lymph-node-biopsy-for-conjunctival-malignant-melanoma-surgica-peer-reviewed-fulltext-article-OPTH	General	9.4	13.4	13.3	9.8	12.9	11.8	49.6	10.5
43	http://www.archivesofpathology.org/doi/pdf/10.1043/2009-0502-RAR.1?code=coap-site	General	12	16.6	14.2	12.2	15.4	14.1	37.8	8.6
44	https://www.dermnetnz.org/topics/sentinel-lymph-node-biopsy/	Specialty	11.5	14.6	11.4	11.1	13.9	12.5	46.4	7.2
45	http://www.nuclmed.gr/wp/wp-content/uploads/2015/06/11.pdf	General	4.8	9.7	9.2	5.8	9.4	7.8	69.9	9.7
46	https://oatext.com/Thin-melanoma-and-sentinel-lymph-node-biopsy-A-difficult-relationship-between-them.php	General	14	17.9	15.1	13.4	16	15.3	25.9	8.9
47	http://www.eanm.org/content-eanm/uploads/2016/11/2015_EANM_Lymphoscin_SentinelNode.pdf	General	10	13.7	12.8	9.4	13.2	11.8	44.8	8
48	http://genomel.org/wp-content/uploads/Option-Grid-Sentinel-Node-Biopsy-yes-or-no.pdf	General	7.6	12.6	14	9.4	11.4	11	55.1	10.8
49	http://ascopubs.org/doi/full/10.1200/jco.2013.51.8423	General	10.3	13.7	13	10.6	14.1	12.3	47.7	8.7
50	http://jnm.snmjournals.org/content/47/2/191.full	General	9.4	13	13.9	9.7	12.4	11.7	45.9	9.2

**Figure 2:**
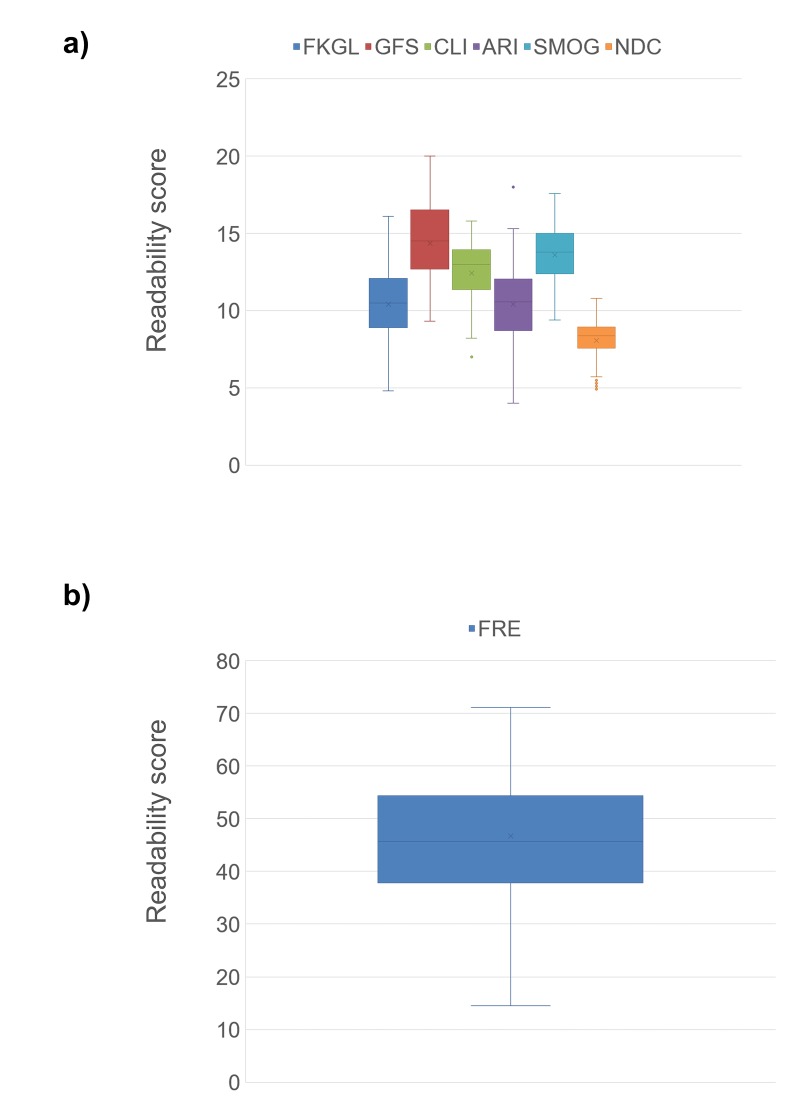
Readability scores of the first 50 websites according to: a) grade level-based assessment formulae and b) the Flesch Reading Ease. FKGL = Flesch-Kincaid Grade Level; GFS = Gunning-Fog Score; CLI = Coleman-Liau Index; ARI = Automated Readability Index; SMOG = Simple Measure of Gobbledygook; NDC = New Dale-Chall Score; FRE = Flesch Reading Ease.

All seven assessment tools reported statistically significant results in that the calculated p-value was less than the standard alpha value of 0.05. In other words, all seven average readability scores (“average” meaning average of the top 50 websites), as calculated by the assessment tools, were significantly different (worse) than their respective “acceptable” readability scores previously outlined in the Methods section. This observation remained unchanged whether or not journal articles, a subgroup that could potentially bias observations towards worse readability scores, were included in the analysis. Distributions of readability scores of the health information websites were outlined in box-and-whisker plots (Figure 2a, 2b). A comparison of mean readability scores between the websites that were classified as “general”, and the websites that were classified as “specialty” is shown in Table 4. Specialty websites tended to have better readability than general websites, but these differences were not statistically significant. A comparison of mean readability scores between peer-reviewed journal articles, and all other websites, is shown in Table 5. Journal articles were significantly less readable than non-journal articles. A comparison of mean readability scores between the top 10 websites that were identified by the search, and the remaining 40 websites, is shown in Table 6. There was no appreciable difference in readability identified by this comparison. Of the 50 websites evaluated, only six (12.0%) were within the limits of the recommended sixth-grade reading level as evaluated by at least one assessment tool, while there were no websites either at or under the sixth-grade reading level according to all seven assessment tools (Table 7). This observation did not change when journal articles were either included or excluded from the analysis.

**Table 4:** Comparison of mean readability scores between general websites and specialty websites. FKGL = Flesch-Kincaid Grade Level; GFS = Gunning-Fog Score; CLI = Coleman-Liau Index; ARI = Automated Readability Index; SMOG = Simple Measure of Gobbledygook; NDC = New Dale-Chall Score; FRE = Flesch Reading Ease.

Readability Measurement	General Websites (n = 40)	Specialty Websites (n = 10)	Mean Difference	P-Value
FKGL	10.6	9.8	0.76	0.19
GFS	14.6	13.5	1.04	0.14
CLI	12.7	11.2	1.56	0.05
ARI	10.7	9.4	1.32	0.12
SMOG	13.7	13.2	0.56	0.20
Average Grade Level	12.5	11.4	1.04	0.12
FRE	45.4	51.8	6.35	0.08
NDC	8.3	7.2	1.10	0.01

**Table 5:** Comparison of mean readability scores between journal articles and non-journal articles. FKGL = Flesch-Kincaid Grade Level; GFS = Gunning-Fog Score; CLI = Coleman-Liau Index; ARI = Automated Readability Index; SMOG = Simple Measure of Gobbledygook; NDC = New Dale-Chall Score; FRE = Flesch Reading Ease.

Readability Measurement	Journal Articles (n = 25)	Non-Journal Articles (n = 25)	Mean Difference	P-Value
FKGL	11.2	9.6	1.60	<0.01
GFS	15.3	13.4	1.90	<0.01
CLI	13.7	11.3	2.42	<0.001
ARI	11.4	9.4	2.04	<0.01
SMOG	14.2	13.0	1.22	<0.01
Average Grade Level	13.2	11.3	1.86	<0.001
FRE	40.3	53.1	12.72	<0.001
NDC	8.8	7.3	1.48	<0.001

**Table 6:** Comparison of mean readability scores between the top 10 websites (which came up in the search) and the remaining 40 websites. FKGL = Flesch-Kincaid Grade Level; GFS = Gunning-Fog Score; CLI = Coleman-Liau Index; ARI = Automated Readability Index; SMOG = Simple Measure of Gobbledygook; NDC = New Dale-Chall Score; FRE = Flesch Reading Ease.

Readability Measurement	Top 10 Websites	Next 40 Websites	Mean Difference	P-Value
FKGL	10.9	10.3	0.60	0.61
GFS	14.7	14.3	0.44	0.35
CLI	12.0	12.5	0.51	0.29
ARI	11.0	10.3	0.71	0.29
SMOG	14.1	13.5	0.54	0.24
Average Grade Level	12.5	12.2	0.35	0.36
FRE	48.1	46.3	1.75	0.36
NDC	7.7	8.2	0.45	0.16

**Table 7:** Distribution of the 50 websites with respect to their ability to meet the sixth-grade reading level guidelines recommended by the United States Department of Health and Human Services. FKGL = Flesch-Kincaid Grade Level; GFS = Gunning-Fog Score; CLI = Coleman-Liau Index; ARI = Automated Readability Index; SMOG = Simple Measure of Gobbledygook; NDC = New Dale-Chall Score; FRE = Flesch Reading Ease.

Readability Measurement	Grade Level			Number of Websites (Overall)	Number of Websites (Journal Articles)	Number of Websites (Non-Journal Articles)
FKGL	Up to Grade 6			2	1	1
	Grade 6-10			18	11	7
	Beyond Grade 10			30	13	17
GFS	Up to Grade 6			0	0	0
	Grade 6-10			4	2	2
	Beyond Grade 10			46	23	23
CLI	Up to Grade 6			0	0	0
	Grade 6-10			10	1	9
	Beyond Grade 10			40	24	16
ARI	Up to Grade 6			4	1	3
	Grade 6-10			18	10	8
	Beyond Grade 10			28	14	14
SMOG	Up to Grade 6			0	0	0
	Grade 6-10			2	1	1
	Beyond Grade 10			48	24	24
FRE	Easy (80-100)			0	0	0
	Average (60-79)			7	1	6
	Difficult (0-59)			43	24	19
NDC	Up to Grade 6			6	0	6
	Grade 6-10			11	3	8
	Beyond Grade 10			33	22	11

## Discussion

Despite readability scores varying between the different formulae utilized, a consistent observation emerged from the analysis. None of the 50 websites were consistently written at a reading level below the sixth-grade level, as recommended by the AMA and USDHHS guidelines, and this observation overwhelmingly highlights a need for major change. The rationale behind these readability guidelines is based upon evidence that the average reading level among American adults is between grades eight and nine, and that approximately one in five of these people read below a grade five level and are considered functionally illiterate [23]. Although according to a report by Statistics Canada, the Canada-United States literacy gap is sizable, with the average Canadian adult more literate than the average American adult by almost a full year of schooling, 48% of Canadian adults 16 years of age or older did not possess the literacy skills required by the current workforce, with many of them being new immigrants [24]. This is especially problematic as literacy is known to be highly positively correlated with socioeconomic status (SES) [25]. Those people with lower literacy rates, and thereby more commonly of lower SES, are also more likely to suffer from a plethora of chronic health conditions that include: type 2 diabetes, hypertension, ischemic heart disease, mental illness, and cancer. Populations with low literacy rates also have many risk factors for these conditions that include: smoking, alcohol and illegal drug use, adverse childhood experiences, and others [26,27]. Interestingly, one study found that the relative risk of developing melanoma was actually decreased in people with lower annual household incomes, but despite this observation those individuals with lower SES were more likely to have a later-stage disease at diagnoses. This observation could potentially be due to substantial barriers that these individuals face when accessing health care (e.g., waitlists in Canada and the lack of universal health care in the United States) [27]. This was an important observation because the prognosis of melanoma is highly dependent on its stage at the time of diagnosis.

Table 4 suggests that there were no significant differences in the readability scores of general medical websites as compared to specialty-specific websites when evaluated by any of the measurements utilized. However, specialty websites tended to be more readable, perhaps because the authors may be experts on the subjects, and are able to better present the relevant information in terms that are more readily comprehended by the general public. Table 5 identifies a significant discrepancy (p-values well below 0.01 across all measures) between the readability of journal articles compared with non-journal articles. This observation is not surprising because journal articles are written for an academic audience that would be expected to have higher literacy scores than the general population. Regardless, these journal articles are identified in the top online search results that are accessible by the general population. Interestingly, articles published in peer-reviewed journals, or links to such articles, represented half of the top 50 search results, most likely because the search phrase was very specific. This suggests that the breadth of online educational resources is actually even worse for people with limited literacy than was previously believed. Table 6 shows that there was no significant difference or trend in the readability scores of the top 10 search results, when compared to the next 40. This is important information because the top 10 websites are much more likely to be accessed by patients, in part because they comprise the first page of search results. The top 10 websites were evenly split between five general websites and five specialty websites. The specialty websites were all authored by organizations or research institutes focused on the study and/or treatment of melanoma (Table 3).

It is important for individuals with either a suspected or confirmed melanoma diagnosis to learn about their cancer, and to understand their recommended management plan, that often includes SLNB. It is expected that members of the patients’ healthcare management team, especially the surgeon, will review with them the risks and benefits of SLNB. However, online resources do still represent an important and readily available source of supplementary educational information; it is crucial that the treating physician is cognizant of website readability when recommending or directing patients toward particular resources. This is the first study to evaluate the readability of online resources for SLNB, and one of few to assess the proportion of journal articles amongst the search results.

The current study has several limitations that must be reviewed. The scope and coverage of available online patient resources may not have been ideal, as only a single search phrase and a single search engine were utilized, and only 50 websites were analyzed in detail. However, we found that variations on the search phrase did not appreciably change the search results, and that Google is currently the most favored search engine for finding health-related information [15], often cited as being the most reliable and least redundant [28]. Furthermore, Google metrics and analytics for the United States suggest that most users evaluate a median of two to four webpages (20-40 search results) for a given search, thus providing a good rationale for our 50-website focus. The study also is limited in the applicability of its observations to more ethnically and geographically diverse patient populations. Only English websites were included and analyzed in the current study. People living in other countries, who may speak other languages, may utilize other search engines that yield different search results, as well as have unique measures or standards of reading literacy. The cross-sectional nature of our study must also be considered, as the Internet is dynamic and is constantly evolving. The same search performed even months earlier or later would have likely yielded somewhat different results; in fact, a repeat search was performed on November 6th, 2017, and the list of top 50 websites the search yielded was 86% similar to the original list. Despite these limitations, we intended this study to inform others about the current state of online patient resource readability, and to provide suggestions that could help facilitate much-needed change.

The observation that the websites’ readability scores were inadequate amongst multiple measures suggests that complex sentence structure, semantics, and vocabulary are all key issues that must all be addressed in the future. When editing or developing online resources, medical jargon must also be avoided whenever possible. This is especially crucial in the first few lines of text, or in any sort of overview paragraph, because if a user is unable to initially comprehend a resource they may cease reading altogether [29]. If using medical terminology is unavoidable, it would be helpful and intuitive to add either a hyperlink to an understandable definition, or a mouse-over pop-up box that contains the definition [30]. Alternatively, dictionary, thesaurus or translation support could be provided, though this would require more time and effort by the user [30]. Keywords and phrases should also be highlighted and emphasized [30]. Educational objectives should be targeted at the sixth-grade reading level, and should also be carefully reviewed so that the authors have a better understanding of the most appropriate language structures to utilize. If re-writing a resource is necessary, authors should focus on utilizing plainspoken words, simple sentence structures, and the active voice, whenever possible. Other areas for future study include investigating whether the timing and character of patient internet searches (e.g., before versus after meeting with the treating surgeon) is impacted by preoperative counselling. Projects such as Health on the Net Foundation show promise in addressing other important dimensions of health information accessibility, such as accuracy and quality, which were not the focus of the current study.

The findings of the current study are consistent with several other reports that have evaluated the readability of online patient educational resources for many different medical procedures and conditions. In 2014, Vargas et al. evaluated 102 articles for patient-directed content obtained from the top 12 websites found with the Google search terms “hernia repair surgery”, as well as the top 10 articles from each of the top 12 consumer magazines (comparison group), and found that all of the 102 articles identified were above the recommended sixth-grade reading level [8]. Jayaweera and Zoysa’s 2016 report assessed the top 50 hits from several different internet search engines (for the search terms “laparoscopic cholecystectomy”). After removing duplicates, they analyzed the resources identified for usability, accessibility, reliability and readability (using FRE and GFI, among other tools) [9]. They found that the websites varied greatly in all of the domains they examined, but were especially poor in reliability. In terms of readability, in this study the average FRE and GFI scores identified major shortcomings, though there were some websites that did meet the AMA/USDHHS guidelines’ standards [9]. Kher et al.’s 2017 study identified the top 100 Google hits using the search terms “congestive heart failure”, and analyzed 70 webpages that met study selection criteria for readability using six of the seven tools that we used in the current study [11]. They found that only five of the 70 websites had readability that was either at or below the sixth-grade reading level. All three of these recent studies highlighted the importance of improving the readability of internet-based patient-directed healthcare information resources, and are part of a growing body of literature.

## Conclusions

Overall, we recommend that current and future patient educational online resources should not only be regularly monitored, evaluated, updated and edited by their authors for the accuracy of factual content, with no single website representing the sole authority, but also for their readability, with the ultimate overall goal being improvement of patient accessibility and education through awareness and adherence to current guidelines.
